# Immunogenicity and Safety Results of a Randomized, Three-Arm, Phase IV Clinical Trial of Concomitant Administration of Typhoid Vi Conjugate Vaccine with Measles and Rubella Vaccine in Healthy Infants

**DOI:** 10.3390/v17091237

**Published:** 2025-09-12

**Authors:** Songa Narayana Rao, Deepali Ambike, Mahantesh Patil, Sanjay Vasant Mankar, Nishant Verma, Neeta Hanumante, Lisa Sarangi, Monjori Mitra, Godatwar Preeti, Bhaskar Jedhe Deshmukh, Girish Nanoti, Mohammad Moonis Akbar Faridi, Pavankumar Daultani, Ravindra Mittal, Kapil Maithal, Kevinkumar Kansagra, Deven V. Parmar, Radhakrishnan Kunnathamman, Manickam Elaiyaraja, Trayambak Dutta, Manish Mahajan, Samir Desai

**Affiliations:** 1 Department of Pediatrics, Andhra Medical College and King George Hospital, Visakhapatnam 530002, India; narayanarao.kghamc@gmail.com; 2 Department of Pediatrics, PCMC’s PGI and Yashwantrao Chavan Memorial Hospital, Pimpri-Chinchwad, Pune 411018, India; ambikedeepa@gmail.com; 3 Department of Pediatrics, J N Medical College, KLE Academy of Higher Education and Research, Belagavi 590010, India; drmahantesh@gmail.com; 4 Department of Pediatrics, Mankar Hospital, Pune 411051, India; drmankarsanjay.pulse@gmail.com; 5 Department of Pediatrics, King George’s Medical University, Lucknow 226003, India; nishantverma@kgmcindia.edu; 6 Department of Pediatrics, Bharati Vidyapeeth Medical College & Hospital, Pune 411043, India; neeta.hanumante@gmail.com; 7 Department of Community Medicine, Hi-Tech Medical College & Hospital, Bhubaneswar 751025, India; sarangilisa@gmail.com; 8 Department of Pediatrics, Institute of Child Health, Kolkata 700017, India; monjorimr@gmail.com; 9 Department of Pediatrics, Niloufer Hospital, Hyderabad 500004, India; drpreetinagaraj@gmail.com; 10 Baramati Hospital Private Ltd., Baramati 413102, India; jedhedeshmukh.12@gmail.com; 11 Department of Pediatrics, NKP Salve Institute of Medical Sciences and Lata Mangeshkar Hospital, Nagpur 440019, India; gir655uv@yahoo.co.in; 12 Department of Pediatrics, Era’s Lucknow Medical College and Hospital, Lucknow 22600, India; mmafaridi@yahoo.co.in; 13 New Product Development, Zydus Lifesciences Ltd., Ahmedabad 382481, India; dr_ravindra62@yahoo.com; 14 Vaccine Technology Centre, Zydus Lifesciences Ltd., Ahmedabad 382481, India; kapil.maithal@zyduslife.com (K.M.); m.n.elaiyaraja@zyduslife.com (M.E.); 15 Clinical R&D, Zydus Lifesciences Ltd., Ahmedabad 382481, India; kevinkumarkansagra@zyduslife.com (K.K.); dparmar@zydustherapeutics.com (D.V.P.); 16 Vaccine R&D, Vaccine Technology Centre, Zydus Lifesciences Ltd., Ahmedabad 382481, India; radhakrishnan.k@zyduslife.com; 17 Medical Affairs—BU Biologics, Zydus Lifesciences Ltd., Ahmedabad 382481, India; manish.mahajan@zyduslife.com (M.M.); trayambak.dutta@zyduslife.com (T.D.); 18 BU Biologics & Vaccines, Zydus Lifesciences Ltd., Ahmedabad 382481, India; samirdesai@zyduslife.com

**Keywords:** typhoid conjugate vaccine, measles–rubella vaccine, concomitant vaccination, immunogenicity, seroconversion, public health vaccination

## Abstract

Typhoid fever, measles, and rubella continue to contribute significantly to childhood morbidity and mortality in India. In line with WHO recommendations for co-administration of Typhoid Conjugate Vaccine (TCV) and measles–rubella (MR) vaccine at 9 months of age, this phase IV, randomized, open-label, multicenter clinical trial was conducted to assess their immunological compatibility and safety when administered concomitantly. A total of 900 healthy Indian infants aged 9–10 months were randomized into three groups: Group A received TCV and MR vaccine concomitantly; Group B received MR on Day 0 and TCV on Day 28; Group C received TCV on Day 0 and MR on Day 28. Subjects were followed for 6 months after concomitant/last vaccination. Seroconversion rates (SC) in Groups A/B/C at Day 28 were 90.2%/75.3%/89.5% for anti-Vi; 80.4%/75.2%/77.7% for anti-measles, and 87.7%/84.0%/85.2% for anti-rubella antibodies. By study end, SC for anti-Vi was 87.1%/71.6%/83.0%, while SC for anti-measles and anti-rubella reached ~90% and ≥98%, respectively, across all groups. Geometric mean titers increased significantly for all antigens, with no evidence of immunological interference. Safety assessments showed adverse events in 23.9%/32.0%/32.7% participants in Group A/B/C. Most adverse events were mild, and only one serious adverse event was reported. These findings support the co-administration of TCV and MR vaccine as a safe and effective strategy.

## 1. Introduction

Typhoid fever, a systemic infection caused by *Salmonella enterica* serovar Typhi, measles, and rubella, both viral diseases, preventable by vaccination, continue to pose significant public health challenges [1]. In 2017, typhoid and paratyphoid fevers accounted for an estimated 14.3 million global cases and 135,900 deaths, with a substantial disease burden of 9.8 million disability-adjusted life years [2]. India accounted for more than half of the estimated 14.3 million global typhoid cases in 2017, with children under 15 years disproportionately affected. Recent cohort studies have reported annual typhoid incidence as high as 1173 per 100,000 child-years in Vellore and 576 per 100,000 in Delhi among children aged 6 months to 14 years, with nearly 25% of cases occurring in children under five years of age [3,4]. Measles, among the most contagious viral diseases, caused an estimated 107,500 deaths globally in 2023, primarily affecting unvaccinated children under five years of age [5]. In India, measles and rubella together contribute significantly to childhood morbidity and mortality, with measles alone being responsible for approximately 49,000 child deaths annually, and in recent years reported case rates of measles and rubella were 10.4 per million and 2.3 per million, respectively [6]. Rubella incidence in India has declined due to expanded vaccination, dropping from 2.3 to 1.2 cases per million between 2017 and 2021, with a further 17% reduction reported in 2024 compared to 2023 [7].

To combat the burden of typhoid and measles–rubella, World Health Organization (WHO) recommends early childhood immunization with the Typhoid Conjugate Vaccine (TCV)—a T cell dependent protein–polysaccharide vaccine—and the measles–rubella (MR) vaccine, a live-attenuated formulation that provides durable immunity [8,9]. Current immunization schedules in India recommend administration of TCV from 6 months of age, with routine use typically beginning at 9 months, often alongside the first dose of the MR vaccine, which is scheduled between 9 and 12 months [6,10]. The CDC recommends simultaneous administration of age-appropriate vaccines when no contraindications exist.

Given the alignment of schedules, the WHO recommends co-administration of TCV and MR vaccines at 9 months of age, provided there is no immunological interference between them [7]. ZyVac TCV, developed by Zydus Lifesciences Ltd., is a WHO-prequalified Typhoid Vi Conjugate Vaccine approved by the Drugs Controller General of India (DCGI) for active immunization against *Salmonella typhi* in individuals aged 6 months to 65 years [11]. Similarly, the ZyVac MR vaccine is DCGI-approved for protection against measles and rubella in children 9 months and older. In line with WHO’s recommendation to evaluate immunological compatibility when TCV is given alongside other vaccines, this study was undertaken to assess the non-interference and safety of co-administered TCV and MR vaccines in healthy Indian infants.

## 2. Materials and Methods

### 2.1. Study Design

This was a prospective, randomized, parallel-group, open-label, multicenter, phase IV clinical trial designed to evaluate the immunological non-interference of TCV when co-administered with MR vaccine in healthy infants. The study was conducted from March 2022 to February 2023.

### 2.2. Study Objectives

The primary objective of this study was to evaluate the immunological non-interference of the co-administration of TCV with MR vaccine in healthy infants aged 9 to 10 months. This was assessed by measuring seroconversion rates and geometric mean titers (GMTs) of anti-Vi, anti-measles, and anti-rubella IgG antibodies following vaccination. The secondary objective was to assess the safety of the co-administration of TCV and MR vaccines by monitoring the incidence of solicited local and systemic adverse events (AEs), unsolicited AEs, and serious AEs (SAEs) throughout the study period.

### 2.3. Study Population

**Figure 1:**
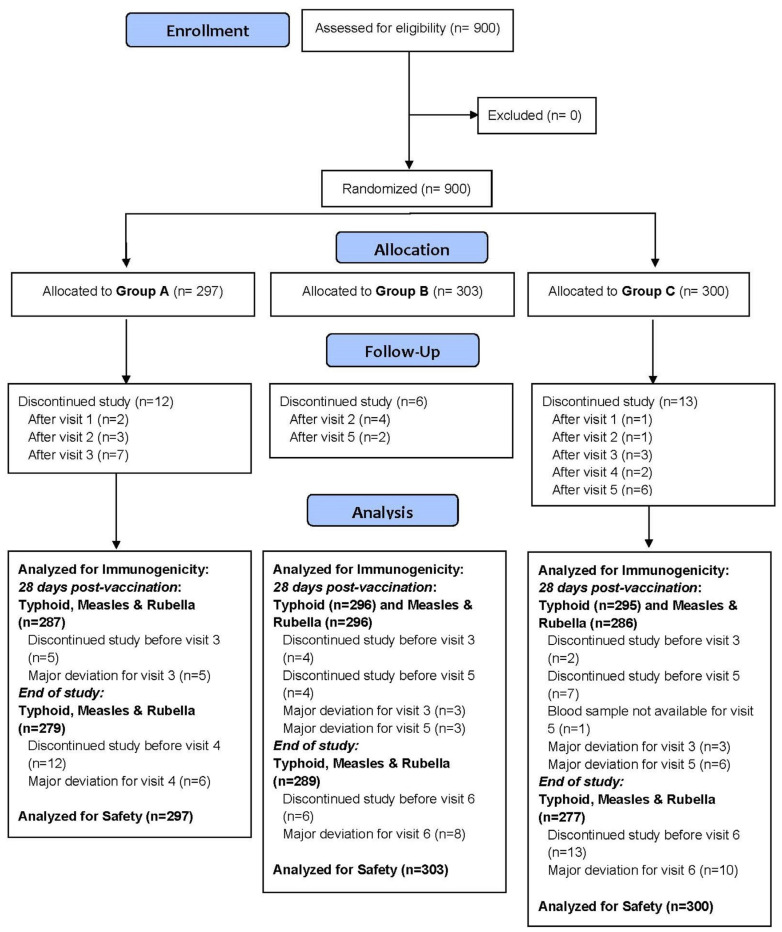
Participant disposition in the study.

A total of 900 healthy infants, aged 9 to 10 months, were enrolled from multiple geographically distributed clinical sites across India. The infants were randomized in a 1:1:1 ratio into three parallel arms using a centralized computer-generated randomization plan (Figure 1). Group A (test group) received the TCV and MR vaccine concomitantly on Day 0. Group B received the MR vaccine on Day 0 followed by TCV on Day 28, while Group C received TCV on Day 0 followed by MR vaccine on Day 28.

#### 2.3.1. Inclusion Criteria

Subjects eligible for enrollment were healthy infants of either gender, aged between 9 and 10 months (age calculated as per completed months). Participants had to be in good general health, as confirmed by medical history and physical examination conducted by the investigator. Additionally, there had to be no history of prior vaccination against typhoid fever, measles, or rubella. Written informed consent was obtained from each subject’s parent, who was also required to be literate and capable of completing the diary card provided for AE recording post-vaccination.

#### 2.3.2. Exclusion Criteria

Infants were excluded from participation if they had a history of hypersensitivity to any component of the study vaccines, or if there was a past or suspected infection with measles, rubella, or *Salmonella typhi*. Other exclusion factors included exposure to these pathogens within 30 days prior to enrollment, febrile illness (body temperature ≥ 37.5 °C), any vaccination within the preceding 30 days, or any clinically significant systemic disorder such as cardiovascular, respiratory, neurologic, gastrointestinal, hepatic, renal, hematologic, or immunological conditions. Subjects were also excluded if they had confirmed or suspected immunodeficiency, were on immunosuppressive or immunostimulant therapy, had a history of thrombocytopenia or bleeding disorders, had recently received blood products or immunoglobulins, or had participated in another clinical study within the last three months.

### 2.4. Vaccine Administration

TCV (batch no. 06FP24B) was available in liquid form and administered as a 0.5 mL single-dose intramuscular (IM) injection into the anterolateral aspect of the upper thigh, following standard aseptic precautions. Each dose contained 25 µg of purified *S. typhi* Vi capsular polysaccharide conjugated to tetanus toxoid. MR vaccine (batch no. 11FM03C) was in lyophilized form and was reconstituted immediately prior to using the accompanying diluent. It was administered as a 0.5 mL single-dose subcutaneous (SC) injection into the anterolateral aspect of the upper thigh, ensuring that it was given in the opposite limb from TCV in cases of concomitant administration.

### 2.5. Study Procedure

All enrolled subjects underwent a structured series of clinical visits, starting with screening and randomization, followed by vaccination and subsequent follow-up visits to systematically evaluate immunogenicity and safety parameters.

At Visit 1 (Day 0), after obtaining written informed consent from a parent, subjects underwent screening procedures including medical history, physical and systemic examinations, and eligibility assessment. Baseline blood samples (3 mL) were collected prior to vaccination for immunological assessment. Vaccines were administered as per group assignment: subjects in Group A received both TCV and MR vaccine concomitantly; subjects in Group B received the MR vaccine; and subjects in Group C received the TCV. Following vaccine administration, each subject was observed on-site for at least 30 min for any immediate hypersensitivity or adverse reactions. Visit 2 (Day 14) involved a follow-up physical examination and review of the diary card. Any AEs reported or observed were documented, and concomitant medication use, if any was recorded. At Visit 3 (Day 28), a second blood sample was collected to assess immunogenicity.

For Group A (TCV + MR), the final visit occurred at month 6 (Visit 4), at which a third blood sample was collected, and a final safety evaluation was performed. For subjects in Group B (MR/TCV) or Group C (TCV/MR), the second vaccine was administered at visit 3, followed by the same 30 min observation period and issuance of a second diary card to record post-vaccination events. For Groups B and C, two additional follow-up visits were conducted. At Visit 4 (Day 14 post-second vaccination) safety assessment was conducted and at Visit 5 (Day 28 post-second vaccination), safety assessment and immunogenicity blood sampling were performed. The final visit for these groups was conducted at month 6 post-second vaccination (Visit 6), which included a final blood draw for immunogenicity and documentation of any SAEs or medically attended AEs (MAAEs).

Throughout the study, unscheduled visits were permitted to address AEs or other clinical concerns. All blood samples were processed for serum separation and stored at −20 °C (±10 °C) before transfer to the central laboratory for analysis. Immunogenicity assessments were scheduled in alignment with the timing of vaccine administration for each study group. For anti-Vi IgG (typhoid), antibody titers were measured at three time points: baseline (prior to TCV administration), 28 days post-TCV, and at month 6/7 post-TCV. These corresponded to Visits 1, 3, and 4 for Group A; Visits 3, 5, and 6 for Group B; and Visits 1, 3, and 6 for Group C. For anti-measles and anti-rubella IgG antibodies, measurements were taken at baseline (prior to MR administration), 28 days post-MR, and at month 6/7 post-MR. These time points were Visits 1, 3, and 4 for Group A; Visits 1, 3, and 6 for Group B; and Visits 3, 5, and 6 for Group C.

### 2.6. Immunogenicity Assessment

Immunogenicity was evaluated by measuring serum IgG antibodies specific to the Vi polysaccharide of *S. typhi*, measles virus, and rubella virus, collected at predefined time points across the study groups. Serum anti-Vi IgG antibodies were quantified using the VaccZyme™ ELISA kits (The Binding Site Group Ltd., Birmingham, UK). Anti-measles and anti-rubella IgG antibodies were measured using NovaLisa^®^ ELISA kits (NovaTec Immundiagnostica GmbH, Dietzenbach, Germany). The seroconversion rate for anti-Vi IgG antibodies was defined as the proportion of subjects achieving a ≥4-fold rise in antibody titer post-vaccination compared to the baseline value [12]. For measles, a subject was considered seroconverted if they were seronegative at baseline (IgG titer < 9 NTU) and achieved a post-vaccination titer ≥ 9 NTU. For rubella, seroconversion was defined as a rise from a baseline level of <10 IU/mL to ≥10 IU/mL post-vaccination. Subjects who were positive for anti-measles and anti-rubella IgG antibodies prior to administration of MR vaccine (antibody titer of ≥9 NTU and ≥10 IU/mL, respectively) were excluded from the analysis. In addition to seroconversion, GMTs of antibodies were calculated prior to and 28 days after administration of the respective vaccines, as well as at study end, and compared across study groups.

### 2.7. Safety Assessment

Following each vaccination, subjects were observed on-site for at least 30 min to monitor for any immediate hypersensitivity or adverse reactions. Parents were provided with diary cards to record any AEs that occurred at home. Solicited local AEs (including pain, redness, swelling, and induration at the injection site) were recorded for 7 days post-vaccination, while solicited systemic AEs (such as fever, rashes, vomiting, diarrhea, cough, running nose, loss of appetite, drowsiness, irritability, and abnormal crying) and unsolicited AEs were documented for 14 days. The completed diary cards were collected back and reviewed by the investigators during subsequent follow-up visits.

### 2.8. Statistical Analysis

At least 238 subjects in the test group and 238 subjects each in both the reference groups were required to achieve the non-inferiority of the test group as compared to the reference groups at 90% power and at 2.5% one-sided level of significance, assuming 87% subjects with seroconversion at 28 days after vaccination with no difference between the test group and the reference groups (Group A—Group B and Group A—Group C), and considering the non-inferiority margin of −10%. Considering that around 10% subjects will be already baseline seropositive and around 10% dropout rate, 900 subjects (includes 20% additional subjects) were required to be enrolled in the study with 1:1:1 allocation ratio. 

Immunogenicity was evaluated by comparing seroconversion rates and geometric mean titers (GMTs) for anti-Vi, anti-measles, and anti-rubella IgG antibodies. Seroconversion rates were compared across groups using Fisher’s exact test. Non-inferiority for co-administration was established if the lower bound of the 95% confidence interval (CI) for the difference in seroconversion rates (Group A vs. Groups B and C) exceeded the −10% margin. Geometric mean titers (GMTs) were analyzed on log-transformed data using unpaired *t*-tests for between-group comparisons and paired *t*-tests for within-group changes. Baseline demographics were summarized descriptively. Continuous variables were compared using unpaired *t*-tests, and categorical variables using Fisher’s exact test. Safety data included all participants receiving at least one vaccine dose and were summarized descriptively by frequency and severity; comparisons were made using Fisher’s exact test.

### 2.9. Ethical Considerations

The clinical trial was approved by Drug Controlled General—India before initiation of enrollment. The trial was also approved by Institutional Ethics Committees of all the participating study sites. The trial was registered with the Clinical Trials Registry India (CTRI) under the registration number CTRI/2022/02/040274 (registered on 14 February 2022). Written informed consent was obtained from parents of all the participants before performing any study related procedures.

## 3. Results

The mean age of participants was comparable across all three groups, with a mean age of 9.2 ± 0.4 months. Similarly, other baseline clinical parameters such as weight, length, temperature, and vital signs showed no statistically significant differences across the groups (Table 1). Participant disposition in the study is presented in Figure 1.

### 3.1. Immunogenicity Outcomes

#### 3.1.1. Seroconversion Rates for Anti-Vi IgG

On Day 28, Group A (TCV + MR) showed a seroconversion rate of 90.2%, which was significantly higher than that of Group B (MR/TCV, 75.3%) and comparable to that of Group C (TCV/MR, 89.5%). This trend persisted till study end, with Group A maintaining 87.1% seroconversion, versus 71.6% in Group B and 83.0% in Group C. The co-administration group (A) thus demonstrated immunological non-inferiority to the sequential schedules, with sustained antibody responses over six months (Table 2).

#### 3.1.2. Seroconversion for Anti-Measles IgG Antibodies

Seroconversion rates on Day 28 were 80.4% in Group A, 75.2% in Group B, and 77.7% in Group C, with no statistically significant differences. By study end, all groups showed similar and high seroconversion (∼90%), confirming that co-administration of TCV with MR does not impair the immunogenicity of the measles component (Table 3).

#### 3.1.3. Seroconversion for Anti-Rubella IgG Antibodies

On Day 28, anti-rubella seroconversion rates were similar across groups (87.7% in Group A vs. 84.0% and 85.2% in Groups B and C, respectively). By study end, nearly all participants had seroconverted, with rates exceeding 98% in all groups. These results support immunological non-inferiority of the co-administration schedule for the rubella component (Table 4).

### 3.2. Geometric Mean Titres of Antibodies

GMTs for anti-Vi, anti-measles, and anti-rubella IgG antibodies were measured at 28 days post-vaccination and at study end (Table 5 and Table 6). Group A (TCV + MR) showed higher anti-Vi GMTs than Group B and was comparable to Group C at both time points. Anti-measles and anti-rubella GMTs were similar across all groups, with titers increasing over time, indicating robust and sustained immune responses. These findings further support the non-inferiority of the co-administration schedule.

### 3.3. Safety Outcomes

The incidence of adverse events (AEs) was lowest in Group A (23.9%), compared to 32.0% in Group B and 32.7% in Group C (Table 7). Most AEs across all groups were mild (Grade 1), accounting for 100% in Group A and 99.5% in Groups B and C. A single serious adverse event (SAE) occurred in Group C, involving a 9-month-old male who experienced a febrile convulsion following TCV administration. The child was hospitalized, treated as per standard pediatric protocol, and recovered fully without sequelae. The SAE was considered related to TCV administration.

**Figure 2:**
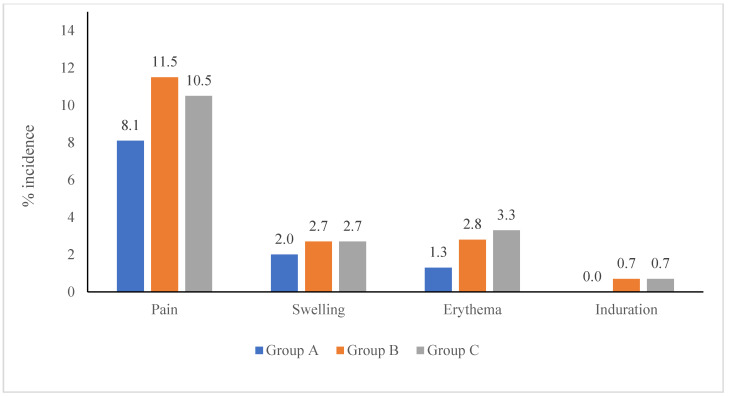
Solicited local adverse events.

**Figure 3:**
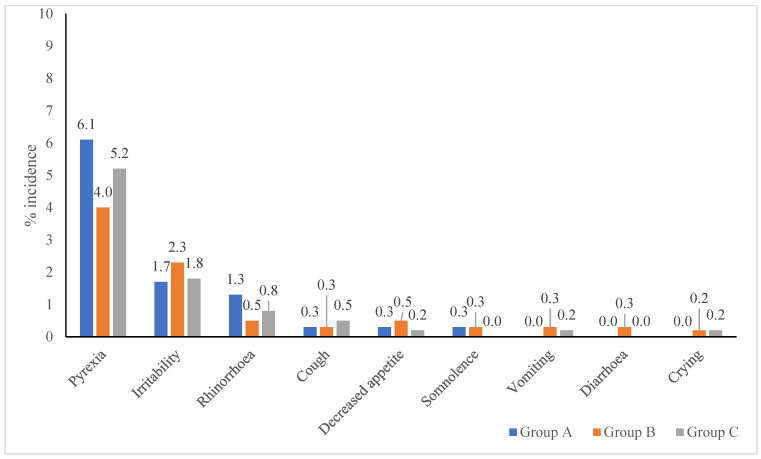
Solicited systemic adverse events.

Solicited AEs were most frequent, comprising 88.3% in Group A, 86.4% in Group B, and 83.4% in Group C. Local AEs were lower in Group A (68 events) than in Group B (106) and Group C (103), with injection site pain being the most common (Figure 2). Group A also reported fewer systemic AEs (30 events) versus 53 events in each of the other groups. Pyrexia was the most common systemic AE across groups (Figure 3). Unsolicited AEs were slightly more frequent in Group C (16.6%) than in Group A (11.7%) and Group B (13.6%), with pyrexia, rhinorrhoea, cough, and diarrhea being the most common (Table 8).

#### 3.3.1. Local Adverse Events by Vaccine Type

Solicited local adverse events were analyzed separately for TCV and MR vaccine administrations to assess individual reactogenicity profiles. Following TCV administration, local AEs were reported in 17.5% of cases in Group A (52/297), 21.1% in Group B (63/299), and 28.0% in Group C (84/300). The most common event was injection site pain. In contrast, following MR vaccine administration, Group A reported fewer local reactions (5.4%, 16/297) than Group B (14.2%, 43/303) and Group C (6.4%, 19/298). Pain at the injection site was the most common symptom, occurring in 3.0% of MR vaccinations in Group A, compared to 8.9% in Group B and 5.0% in Group C (Table 9).

#### 3.3.2. Systemic Adverse Events by Vaccine Type

Following concomitant administration in Group A, 30 solicited systemic events were reported with the most common being pyrexia (6.1%) and irritability (1.7%). In the sequential administration groups, systemic AEs were more often reported following the first vaccine dose compared to the second. In Group B, the MR vaccine (first dose) was followed by 32 solicited systemic events, compared to 21 after the subsequent TCV dose. Similarly, in Group C, the initial TCV dose was associated with 33 events, while the subsequent MR vaccine dose resulted in 20 events. Across all groups, pyrexia remained the most commonly reported systemic AE. Unsolicited systemic AEs were also evaluated (Table 10).

### 3.4. Medically Attended Adverse Events (MAAEs)

A total of 10 MAAEs were reported in Group A, 20 in Group B, and 17 in Group C. All events were mild and self-limiting. Detailed distribution by vaccine and symptom is provided in Table 11.

## 4. Discussion

This phase IV, randomized, open-label clinical trial was conducted to evaluate the immunological non-interference and safety of co-administering TCV with MR vaccine in infants aged 9 to 10 months. The findings from this study demonstrate that concomitant administration of TCV and MR vaccines is immunologically non-inferior and well tolerated, supporting its suitability for inclusion in the national immunization schedule.

In the present study, concomitant administration of TCV and MR vaccines (Group A) resulted in high seroconversion rates for anti-Vi IgG (90.2%) and anti-rubella IgG (87.7%) at 28 days, with sustained antibody levels observed at 6 months. These findings are consistent with the study by Sirima et al. in Burkina Faso, where co-administration of TCV with MR and Yellow Fever vaccines in 9-month-old infants resulted in an anti-Vi IgG seroconversion rate of 87.8%, along with high seropositivity rates for MR antigens. The anti-Vi GMT at 28 days post-vaccination reported in our study (1338.8 U/mL) was within the range reported in their cohort (1203.7 EU/mL), supporting the comparable immunogenic performance of the co-administered schedule [13]. Another Burkina Faso study in older children (15–23 months) by the same group evaluated TCV co-administered with MR and group A meningococcal conjugate vaccine (MCV-A), reporting an anti-Vi seroconversion rate of 96.0% and GMTs of 3707.3 EU/mL. Although conducted in an older age group, the findings support the absence of immunological interference when TCV is administered with other EPI vaccines [14].

The immunological characteristics of the co-administered vaccines help explain the favorable outcomes observed. The MR vaccine is a live-attenuated formulation that transiently replicates in the host, stimulating both humoral and cellular immunity through activation of CD8^+^ cytotoxic T cells and balanced CD4^+^ Th1/Th2 responses, while also acting as its own adjuvant via innate immune activation [15]. In contrast, TCV is an inactivated polysaccharide–protein conjugate vaccine formulated with an aluminum adjuvant, which promotes strong antibody (Th2-type) responses but provides limited cellular immune stimulation. This difference likely underlies the higher anti-Vi seroconversion rates and GMTs observed in the concomitant arm compared with the MR-first sequential arm, as the robust cellular milieu induced by MR may have facilitated more effective priming of Vi-specific responses, whereas sequential administration after MR may coincide with transient innate immune modulation that dampens subsequent TCV responses. A similar pattern was also observed in a clinical study by Vadrevu KM et al., which evaluated concomitant administration of another TCV with measles vaccine in infants [16]. In that study, GMTs of anti-Vi antibodies in subjects who received measles vaccine at 9 months of age and TCV 28 days later were roughly half of those observed in subjects who received both vaccines concomitantly at the same age, further supporting the immunological advantage of simultaneous administration [16]. Reports of interference between TCV and other inactivated conjugates such as meningococcal A vaccine are more plausibly attributed to competition for shared T-helper pathways or aluminum adjuvant-driven innate signaling rather than to antigenic overlap [17]. These observations emphasize that interference is context-dependent: while inactivated–inactivated combinations may compete immunologically, the strong cellular immunity elicited by live MR vaccination appears to support rather than hinder concomitant TCV administration.

Comparable immunogenic outcomes were reported in a Nepalese phase III trial by Saluja et al., which assessed Vi-DT co-administered with the MMR vaccine in infants aged 9–15 months [18]. That study reported 100% anti-Vi seroconversion and high seropositivity for measles (90.4%) and rubella (93.8%). In our study, anti-Vi seroconversion was 90.2%, and seroconversion for measles and rubella were 80.4% and 87.7%, respectively. A separate nested substudy conducted in Malawi by Nampota-Nkomba et al. evaluated Typbar TCV and MR co-administration in infants aged 9–11 months, reporting 99.0% anti-Vi seroconversion with GMTs of 2594.8 EU/mL [19]. Seroconversion for MR antigens was also consistent with our findings, with 83.5% for measles and 75.3% for rubella. Across all studies, the data indicate that co-administration of TCV with MR/MMR vaccines does not interfere with immune responses to the target antigens, regardless of study location, vaccine brand, or immunization schedule. In our study, Group B first received MR vaccine followed by TCV after a 28-day interval; this group showed lower anti-Vi seroconversion rates and GMTs compared to the concomitant and reverse-sequence arms. This tempered response to TCV in Group B may be attributed to transient immune modulation following live-attenuated MR vaccination, which could temporarily dampen the innate or adaptive priming environment required for optimal response to subsequently administered TCV [20].

AEs following vaccination were predominantly mild and aligned with established reactogenicity profiles of TCV and MR vaccines. Local adverse events occurred more frequently after TCV administration, with pain, swelling, and erythema at the injection site being the most commonly reported symptoms. These reactions are characteristic of polysaccharide–protein conjugate vaccines and likely result from localized innate immune activation [21]. Among the study groups, local AEs after TCV were most frequent in Group C (28.0%), followed by Group B (21.1%) and Group A (17.5%). In contrast, MR vaccine administration was associated with lower local AE rates, particularly in Group A (5.4%), consistent with the typically milder local reactogenicity of live-attenuated vaccines such as MR vaccine. Systemic adverse events, including pyrexia, irritability, rhinorrhoea, and cough, were reported after both TCV and MR vaccines, with pyrexia being the most common across all groups. These symptoms are well-recognized post-vaccination responses, attributed to transient cytokine release and immune activation, especially after primary antigen exposure. In the sequential administration arms (Groups B and C), systemic AEs were more frequent after the first dose, irrespective of whether it was TCV or MR, suggesting a more pronounced innate response during initial immune priming. Importantly, in the co-administration group (Group A), systemic AE rates remained low (10.1%), indicating that concurrent delivery of TCV and MR did not lead to increased systemic reactogenicity.

This safety profile is consistent with findings from other co-administration studies. In our study, 23.9% of subjects in Group A reported adverse events, all mild in nature. One serious adverse event (febrile convulsion) was reported in Group C and was attributed to TCV administration. Similarly, Sirima et al. reported 8.2% systemic AEs in infants receiving TCV with MR and YF vaccines at 9 months, and a higher but still mild AE frequency (61–69%) in a 15–23-month cohort receiving TCV with MR and MCV-A [13,14]. In the Nepalese trial, solicited AEs were reported in 6.1% (Vi-DT + MMR group) and 5.0% (MMR-only group) participants, with no serious events [18]. The Malawi study also reported 9% solicited systemic AEs and 24% unsolicited AEs, predominantly mild and transient [19].

The results of this phase IV study provide evidence that the co-administration of TCV and MR vaccines at 9–10 months of age is immunologically non-inferior to sequential administration, with robust antibody responses and an acceptable safety profile. While anti-measles and anti-rubella responses were comparable across groups, anti-Vi seroconversion rates and GMTs were significantly higher in the co-administration group (A) compared to the MR-first group (B), indicating a more favorable response when TCV is administered first or concurrently. The study’s multicentric design, large sample size, and long follow-up period strengthen the generalizability of its findings. However, certain limitations should be acknowledged. The open-label nature of the trial could introduce reporting bias in adverse event monitoring, although standardized diary cards and investigator assessments were used to mitigate this risk.

## 5. Conclusions

Concomitant administration of TCV and MR vaccines in infants aged 9–10 months demonstrated robust immunogenicity and a favorable safety profile, with no evidence of immunological interference. These findings support the integration of co-administered TCV and MR vaccines into routine immunization schedules, enhancing operational efficiency and vaccine coverage in national public health programs targeting early childhood infectious disease prevention.

## Figures and Tables

**Table 1:** Baseline demographic parameters across different groups.

Parameter		Group A (TCV + MR) [*N* = 297]	Group B (MR/TCV) [*N* = 303]	Group C (TCV/MR) [*N* = 300]	*p* Value (A vs. B)	*p* Value (A vs. C)
Age (months) *		9.2 ± 0.4	9.2 ± 0.4	9.2 ± 0.4	0.43	0.58
Gender ^#^	Male	161 (54.2%)	153 (50.5%)	161 (53.7%)	0.37	0.93
	Female	136 (45.8%)	150 (49.5%)	139 (46.3%)		
Length (cm) *		68.9 ± 6.4	69.3 ± 6.5	69.2 ± 6.2	0.47	0.51
Weight (kg) *		8.2 ± 1.0	8.1 ± 1.1	8.2 ± 1.1	0.74	0.54
Temperature (°C) *		36.8 ± 0.3	36.8 ± 0.3	36.8 ± 0.3	0.24	0.49
Heart Rate (/min) *		112.2 ± 11.1	112.3 ± 10.1	113.7 ± 10.4	0.87	0.08
Respiratory Rate (/min) *		32.7 ± 6.3	32.9 ± 6.2	33.0 ± 5.6	0.68	0.58

A vs. B = Group A vs. Group B; A vs. C = Group A vs. Group C. * Data presented as mean ± SD. ^#^ Data presented as n (%).

**Table 2:** Seroconversion rate for anti-Vi IgG across different groups at Day 28 and at the end of the study.

Study Group	Seroconversion Rate *	Group A–Group B or C ^#^	*p* Value
Seroconversion Rate at Day 28			
Group A [*N* = 287]	259 (90.2%)	NA	NA
Group B [*N* = 296]	223 (75.3%)	14.9% (8.6%, 21.1%)	<0.0001 ^$^
Group C [*N* = 295]	264 (89.5%)	0.8% (–4.5%, 6.0%)	0.79 ^@^
Seroconversion Rate at the end of the study			
Group A [*N* = 287]	243 (87.1%)	NA	NA
Group B [*N* = 296]	207 (71.6%)	15.5% (8.6%, 22.2%)	<0.0001 ^$^
Group C [*N* = 295]	230 (83.0%)	4.1% (−2.1%, 10.3%)	0.19 ^@^

NA = Not applicable. * Data presented as no. (%). ^#^ Data presented as % (95% CI). ^$^ Fisher’s exact test; Group A vs. Group B. ^@^ Fisher’s exact test; Group A vs. Group C.

**Table 3:** Seroconversion rate for anti-measles IgG antibodies across different groups on Day 28 and at the end of study.

Study Group	*n* ^	Seroconversion Rate *	Group A–Group B or C #	*p* Value
Seroconversion rate on Day 28				
Group A [*N* = 287]	271	218 (80.4%)	NA	NA
Group B [*N* = 296]	278	209 (75.2%)	5.3% (−2.0%, 12.4%)	0.15 ^$^
Group C [*N* = 286]	238	185 (77.7%)	2.7% (−4.6%, 10.1%)	0.51 ^@^
Seroconversion rate at the end of study				
Group A [*N* = 287]	263	237 (90.1%)	NA	NA
Group B [*N* = 296]	271	244 (90.0%)	0.1% (−5.4%, 5.5%)	1.0 ^$^
Group C [*N* = 286]	228	206 (90.4%)	−0.2% (−6.0%, 5.3%)	1.0 ^@^

NA = Not applicable. ^ Data presented as no. of subjects seronegative prior to vaccination. * Data presented as no. (%). # Data presented as % (95% CI). ^$^ Fisher’s exact test; Group A vs. Group B. ^@^ Fisher’s exact test; Group A vs. Group C.

**Table 4:** Seroconversion rate for anti-rubella IgG antibodies across different groups on Day 28 and at the end of the study.

Study Group	*n* ^	Seroconversion Rate *	Group A–Group B or C #	*p* Value
Seroconversion rate on Day 28				
Group A [*N* = 287]	261	229 (87.7%)	NA	NA
Group B [*N* = 296]	263	221 (84.0%)	3.7% (−2.6%, 10.0%)	0.26 ^$^
Group C [*N* = 286]	223	190 (85.2%)	2.5% (−3.8%, 9.1%)	0.43 ^@^
Seroconversion rate at the end of study				
Group A [*N* = 287]	254	252 (99.2%)	NA	NA
Group B [*N* = 296]	258	256 (99.2%)	0.0% (−2.4%, 2.4%)	1.0 ^$^
Group C [*N* = 286]	213	210 (98.6%)	0.6% (−1.9%, 3.7%)	0.66 ^@^

NA = Not applicable. ^ Data presented as no. of subjects seronegative prior to vaccination. * Data presented as no. (%). # Data presented as % (95% CI). ^$^ Fisher’s exact test; Group A vs. Group B. ^@^ Fisher’s exact test; Group A vs. Group C.

**Table 5:** GMTs of antibodies 28 days after respective vaccination.

	Group A [*N* = 287]	Group B [*N* = 296]	Group C [*N* = 295]	*p* Value (A vs. B)	*p* Value (A vs. C)
Anti-Vi IgG (U/mL)					
Pre-vaccination	4.8 (4.2, 5.4)	11.5 (9.0, 14.7)	5.1 (4.5, 5.9)	<0.0001	0.42
28 d post-vaccination	1338.8 (1074.1, 1668.7)	749.6 (583.1, 963.6)	1218.4 (971.4, 1528.1)	<0.01	0.56
*p* value	<0.0001	<0.0001	<0.0001	NA	NA
Anti-measles IgG (NTU)					
Pre-vaccination	1.9 (1.8, 2.1)	1.8 (1.7, 2.0)	2.4 (2.2, 2.8)	0.50	<0.01
28 d post-vaccination	12.0 (11.3, 12.8)	11.3 (10.5, 12.2)	11.8 (11.0, 12.6)	0.22	0.69
*p* value	<0.0001	<0.0001	<0.0001	NA	NA
Anti-rubella IgG (IU/mL)					
Pre-vaccination	1.3 (1.1, 1.6)	1.5 (1.2, 1.8)	2.2 (1.8, 2.7)	0.31	<0.001
28 d post-vaccination	26.8 (23.7, 30.3)	24.8 (22.1, 27.8)	26.4 (23.2, 30.0)	0.38	0.88
*p* value	<0.0001	<0.0001	<0.0001	NA	NA

NA = Not applicable. Data presented as geometric mean (95% CI of geometric mean). A vs. B = Group A vs. Group B; A vs. C = Group A vs. Group C.

**Table 6:** GMTs of antibodies at end of the study.

	Group A GMT (*N* = 279)	Group B GMT (*N* = 289)	Group C GMT (*N* = 277)	*p* Value (A vs. B)	*p* Value (A vs. C)
Anti-Vi IgG (U/mL)					
Pre-vaccination	4.8 (4.2, 5.5)	11.8 (9.2, 15.2)	5.1 (4.4, 5.8)	<0.0001	0.61
End of study	90.6 (77.9, 105.5)	111.5 (93.6, 132.9)	90.3 (77.0, 105.9)	0.08	0.97
*p* value	<0.0001	<0.0001	<0.0001	NA	NA
Anti-measles IgG (NTU)					
Pre-vaccination	1.9 (1.8, 2.1)	1.8 (1.7, 2.0)	2.5 (2.2, 2.9)	0.49	<0.01
End of study	16.7 (15.8, 17.7)	16.7 (15.8, 17.8)	16.9 (16.0, 17.9)	0.97	0.75
*p* value	<0.0001	<0.0001	<0.0001	NA	NA
Anti-rubella IgG (IU/mL)					
Pre-vaccination	1.3 (1.1, 1.6)	1.5 (1.2, 1.8)	2.3 (1.8, 2.8)	0.40	<0.001
End of study	89.2 (82.3, 96.8)	75.3 (69.4, 81.7)	77.9 (70.7, 85.7)	<0.01	0.03
*p* value	<0.0001	<0.0001	<0.0001	NA	NA

NA = Not applicable. Data presented as geometric mean (95% CI of geometric mean). A vs. B = Group A vs. Group B; A vs. C = Group A vs. Group C.

**Table 7:** Summary of adverse events.

Parameter	Group A (TCV + MR) [*N* = 297]	Group B (MR/TCV) [*N* = 303]	Group C (TCV/MR) [*N* = 300]
No. (%) of subjects with AEs ^@^	71 (23.9%)	97 (32.0%)	98 (32.7%)
No. of AEs reported *	111	184	187
Adverse Events by Severity Grade ^#^			
Mild (Grade 1)	111 (100.0%)	183 (99.5%)	186 (99.5%)
Moderate (Grade 2)	0 (0.0%)	1 (0.5%)	0 (0.0%)
Severe (Grade 3)	0 (0.0%)	0 (0.0%)	1 (0.5%)
Adverse Events by Association ^#^			
Related—TCV	52 (46.8%)	84 (45.7%)	118 (63.1%)
Related—MR vaccine	21 (18.9%)	75 (40.8%)	39 (20.9%)
Related—Both	25 (22.5%)	0 (0.0%)	0 (0.0%)
Not related	13 (11.7%)	25 (13.6%)	30 (16.0%)
Adverse Events by Reporting (Solicited/Unsolicited) ^#^			
Solicited	98 (88.3%)	159 (86.4%)	156 (83.4%)
Unsolicited	13 (11.7%)	25 (13.6%)	31 (16.6%)
Serious Adverse Events ^#^			
No. of SAEs	0 (0.0%)	0 (0.0%)	1 (0.5%)
No. of patients with SAEs *	0	0	1

A vs. B = Group A vs. Group B; A vs. C = Group A vs. Group C. Data presented as n (%) unless specified. * Data presented as n. ^@^ % calculated from No. of subjects analyzed for safety. ^#^ % calculated from No. of adverse events reported.

**Table 8:** Distribution of unsolicited adverse events.

Parameter	Group A (TCV + MR) [*N* = 297]	Group B (MR/TCV) [*N* = 303]	Group C (TCV/MR) [*N* = 300]	*p* Value (A vs. B)	*p* Value (A vs. C)
No. of unsolicited AEs reported *	13	25	31	NA	NA
Local AEs *	0	0	0	NA	NA
Systemic AEs *	13	25	31	NA	NA
Pyrexia	4 (1.3%)	6 (2.0%)	7 (2.3%)	0.75	0.55
Rhinorrhoea	3 (1.0%)	2 (0.7%)	5 (1.7%)	0.68	0.72
Cough	2 (0.7%)	3 (1.0%)	7 (2.3%)	1.0	0.18
Diarrhea	1 (0.3%)	3 (1.0%)	2 (0.7%)	0.62	1.0
URTI	1 (0.3%)	2 (0.7%)	2 (0.7%)	1.0	1.0
Rhinitis	1 (0.3%)	1 (0.3%)	0 (0.0%)	1.0	0.50
Ear pain	1 (0.3%)	0 (0.0%)	1 (0.3%)	0.50	1.0
Nasopharyngitis	0 (0.0%)	2 (0.7%)	1 (0.3%)	0.5	1.0
Rash	0 (0.0%)	2 (0.7%)	1 (0.3%)	0.50	1.0
Vomiting	0 (0.0%)	2 (0.7%)	0 (0.0%)	0.50	NA
Acarodermatitis	0 (0.0%)	1 (0.3%)	0 (0.0%)	1.0	NA
Dermatitis atopic	0 (0.0%)	1 (0.3%)	0 (0.0%)	1.0	NA
Eczema	0 (0.0%)	0 (0.0%)	1 (0.3%)	NA	1.0
Febrile convulsion	0 (0.0%)	0 (0.0%)	1 (0.3%)	NA	1.0
Gastroenteritis	0 (0.0%)	0 (0.0%)	1 (0.3%)	NA	1.0
Miliaria	0 (0.0%)	0 (0.0%)	1 (0.3%)	NA	1.0
Seborrhoeic dermatitis	0 (0.0%)	0 (0.0%)	1 (0.3%)	NA	1.0

A vs. B = Group A vs. Group B; A vs. C = Group A vs. Group C; NA = Not applicable, URTI = Upper respiratory tract infection. Data presented as n (%) unless specified. % calculated from no. of subjects analyzed for safety. * Data presented as n.

**Table 9:** Solicited local AEs reported after TCV and MR administration.

	Group A (TCV + MR) [*N* = 297]	Group B (MR/TCV) [*N* = 303]	Group C (TCV/MR) [*N* = 300]
**Solicited local AEs reported after TCV administration**			
No. of TCV administration, n	297	299	300
Local AEs, *n*	52	63	84
Pain, *n* (%)	39 (13.1%)	42 (14.0%)	48 (16.0%)
Swelling, *n* (%)	7 (2.4%)	9 (3.0%)	14 (4.7%)
Erythema, *n* (%)	6 (2.0%)	10 (3.3%)	18 (6.0%)
Induration, *n* (%)	0 (0.0%)	2 (0.7%)	4 (1.3%)
**Solicited local AEs reported after MR administration**			
No. of MR vaccine administrations, n	297	303	298
Local AEs, *n*	16	43	19
Pain, *n* (%)	9 (3.0%)	27 (8.9%)	15 (5.0%)
Swelling, *n* (%)	5 (1.7%)	7 (2.3%)	2 (0.7%)
Erythema, *n* (%)	2 (0.7%)	7 (2.3%)	2 (0.7%)
Induration, *n* (%)	0 (0.0%)	2 (0.7%)	0 (0.0%)

**Table 10:** Solicited and unsolicited systemic AEs reported after TCV, MR vaccine, and concomitant administration.

	Group A (TCV + MR) [*N* = 297]	Group B (MR/TCV) [*N* = 303]		Group C (TCV/MR) [*N* = 300]	
	Concomitant (TCV + MR)	First Vaccine (MR)	Second Vaccine (TCV)	First Vaccine (TCV)	Second Vaccine (MR)
Solicited systemic AEs reported after TCV, MR vaccine and Concomitant administration					
No. of vaccinated sub., *n*	297	303	299	300	298
Systemic AEs, *n*	30	32	21	33	20
Pyrexia, *n* (%)	18 (6.1%)	12 (4.0%)	12 (4.0%)	20 (6.7%)	11 (3.7%)
Irritability, *n* (%)	5 (1.7%)	8 (2.6%)	6 (2.0%)	11 (3.7%)	0 (0.0%)
Rhinorrhoea, *n* (%)	4 (1.3%)	3 (1.0%)	0 (0.0%)	0 (0.0%)	5 (1.7%)
Cough, *n* (%)	1 (0.3%)	2 (0.7%)	0 (0.0%)	0 (0.0%)	3 (1.0%)
Decreased appetite, *n* (%)	1 (0.3%)	2 (0.7%)	1 (0.3%)	0 (0.0%)	1 (0.3%)
Somnolence, *n* (%)	1 (0.3%)	2 (0.7%)	0 (0.0%)	0 (0.0%)	0 (0.0%)
Vomiting, *n* (%)	0 (0.0%)	1 (0.3%)	1 (0.3%)	1 (0.3%)	0 (0.0%)
Diarrhea, *n* (%)	0 (0.0%)	2 (0.7%)	0 (0.0%)	0 (0.0%)	0 (0.0%)
Crying, *n* (%)	0 (0.0%)	0 (0.0%)	1 (0.3%)	1 (0.3%)	0 (0.0%)
Unsolicited systemic AEs reported after TCV, MR vaccine and Concomitant administration					
Systemic AEs, *n*	13	3	22	10	21
Pyrexia, *n* (%)	4 (1.3%)	1 (0.3%)	5 (1.7%)	2 (0.7%)	5 (1.7%)
Rhinorrhoea, *n* (%)	3 (1.0%)	0 (0.0%)	2 (0.7%)	2 (0.7%)	3 (1.0%)
Cough, *n* (%)	2 (0.7%)	1 (0.3%)	2 (0.7%)	3 (1.0%)	4 (1.3%)
Diarrhea, *n* (%)	1 (0.3%)	0 (0.0%)	3 (1.0%)	1 (0.3%)	1 (0.3%)
URTI, *n* (%)	1 (0.3%)	0 (0.0%)	2 (0.7%)	1 (0.3%)	1 (0.3%)
Rhinitis, *n* (%)	1 (0.3%)	1 (0.3%)	0 (0.0%)	0 (0.0%)	0 (0.0%)
Ear pain, *n* (%)	1 (0.3%)	0 (0.0%)	0 (0.0%)	0 (0.0%)	1 (0.3%)
Nasopharyngitis, *n* (%)	0 (0.0%)	0 (0.0%)	2 (0.7%)	0 (0.0%)	1 (0.3%)
Rash, *n* (%)	0 (0.0%)	0 (0.0%)	2 (0.7%)	0 (0.0%)	1 (0.3%)
Vomiting, *n* (%)	0 (0.0%)	0 (0.0%)	2 (0.7%)	0 (0.0%)	0 (0.0%)
Acarodermatitis, *n* (%)	0 (0.0%)	0 (0.0%)	1 (0.3%)	0 (0.0%)	0 (0.0%)
Dermatitis atopic, *n* (%)	0 (0.0%)	0 (0.0%)	1 (0.3%)	0 (0.0%)	0 (0.0%)
Eczema, *n* (%)	0 (0.0%)	0 (0.0%)	0 (0.0%)	0 (0.0%)	1 (0.3%)
Febrile convulsion, *n* (%)	0 (0.0%)	0 (0.0%)	0 (0.0%)	1 (0.3%)	0 (0.0%)
Gastroenteritis, *n* (%)	0 (0.0%)	0 (0.0%)	0 (0.0%)	0 (0.0%)	1 (0.3%)
Miliaria, *n* (%)	0 (0.0%)	0 (0.0%)	0 (0.0%)	0 (0.0%)	1 (0.3%)
Seborrhoeic dermatitis, *n* (%)	0 (0.0%)	0 (0.0%)	0 (0.0%)	0 (0.0%)	1 (0.3%)

**Table 11:** MAAEs reported after TCV, MR vaccine, and Concomitant administration.

	Group A (TCV + MR) [*N* = 297]	Group B (MR/TCV) [*N* = 303]	Group C (TCV/MR) [*N* = 300]
No. of vaccinated sub.	297	299	298
Local AEs, *n*	0	0	0
Systemic AEs, *n*	10	20	17
Pyrexia, *n* (%)	3 (1.0%)	5 (1.7%)	4 (1.3%)
Rhinorrhoea, *n* (%)	2 (0.7%)	1 (0.3%)	3 (1.0%)
Cough, *n* (%)	1 (0.3%)	2 (0.7%)	4 (1.3%)
Diarrhea, *n* (%)	1 (0.3%)	3 (1.0%)	1 (0.3%)
URTI, *n* (%)	1 (0.3%)	1 (0.3%)	1 (0.3%)
Rhinitis, *n* (%)	1 (0.3%)	0 (0.0%)	0 (0.0%)
Ear pain, *n* (%)	1 (0.3%)	0 (0.0%)	0 (0.0%)
Nasopharyngitis, *n* (%)	0 (0.0%)	2 (0.7%)	1 (0.3%)
Rash, *n* (%)	0 (0.0%)	2 (0.7%)	0 (0.0%)
Vomiting, *n* (%)	0 (0.0%)	2 (0.7%)	0 (0.0%)
Acarodermatitis, *n* (%)	0 (0.0%)	1 (0.3%)	0 (0.0%)
Dermatitis atopic, *n* (%)	0 (0.0%)	1 (0.3%)	0 (0.0%)
Eczema, *n* (%)	0 (0.0%)	0 (0.0%)	0 (0.0%)
Febrile convulsion, *n* (%)	0 (0.0%)	0 (0.0%)	0 (0.0%)
Gastroenteritis, *n* (%)	0 (0.0%)	0 (0.0%)	1 (0.3%)
Miliaria, *n* (%)	0 (0.0%)	0 (0.0%)	1 (0.3%)
Seborrhoeic dermatitis, *n* (%)	0 (0.0%)	0 (0.0%)	1 (0.3%)

## Data Availability

Data are contained within the article.
